# The Role of Intratumor Heterogeneity in the Response of Metastatic Non-Small Cell Lung Cancer to Immune Checkpoint Inhibitors

**DOI:** 10.3389/fonc.2020.569202

**Published:** 2020-12-04

**Authors:** Marcin Nicoś, Paweł Krawczyk, Nicola Crosetto, Janusz Milanowski

**Affiliations:** ^1^ Department of Pneumonology, Oncology and Allergology, Medical University of Lublin, Lublin, Poland; ^2^ Science for Life Laboratory, Department of Medical Biochemistry and Biophysics, Karolinska Institutet, Stockholm, Sweden

**Keywords:** metastatic non-small cell lung cancer (NSCLC), tumor heterogeneity, immunotherapy, tumor mutation burden, tumor microenvironment, neoantigens, programmed-death 1 (PD-1), programmed-death ligand 1 (PD-L1)

## Abstract

Immune checkpoint inhibitors (ICIs) represent one of the most promising therapeutic approaches in metastatic non-small cell lung cancer (M-NSCLC). Unfortunately, approximately 50–75% of patients do not respond to this treatment modality. Intratumor heterogeneity (ITH) at the genetic and phenotypic level is considered as a major cause of anticancer therapy failure, including resistance to ICIs. Recent observations suggest that spatial heterogeneity in the composition and spatial organization of the tumor microenvironment plays a major role in the response of M-NSCLC patients to ICIs. In this mini review, we first present a brief overview of the use of ICIs in M-NSCLC. We then discuss the role of genetic and non-genetic ITH on the efficacy of ICIs in patients with M-NSCLC.

## Immune Checkpoint Inhibitors in Metastatic Non-Small Cell Lung Cancer: An Overview

Understanding the interactions between the immune system and cancer cells has greatly advanced our knowledge of the mechanisms of tumor growth and progression (1). By now, it is clear that the immune system plays a pivotal role not only in eradicating the disease in cancer patients, but also in promoting a long-lasting immunity (2). This key observation has paved the way to the development of immunomodulating agents and opened the era of cancer immunotherapy (3), which culminated with the assignment of the Nobel Price to James P. Allison and Tasuku Honjo in 2018 (4). Modulation of interactions between T-cells, antigen-presenting cells, and tumor cells has helped unleash suppressed immune responses and increase the effective elimination of cancer cells (1, 2).

The availability of immune checkpoint inhibitors (ICIs) has radically changed the management of patients affected by M-NSCLC (3). Commonly used ICIs in these patients include the monoclonal antibodies: nivolumab (5–8), pembrolizumab (9–12), durvalumab (13), atezolizumab (14–16), and avelumab (17), which act by targeting immune checkpoints expressed by tumor infiltrating lymphocytes (TILs)— programmed-death 1 (PD-1)—or expressed by cancer and tumor infiltrating immune cells— programmed-death ligand 1 (PD-L1) (18, 19). The selection of which ICI to use depends on the expression of PD-L1, which can be evaluated using various assays, whose clinical validity has been assessed in numerous clinical trials (5, 6, 8–10, 12–16). ICIs have proven to be better tolerated than standard chemotherapy (2, 6, 10). However, the response to single-agent ICI therapy is not durable, and only a minority of patients have a prolonged benefit (2, 9, 14). Moreover, there is now evidence that dual blockade of CTLA-4 and PD-1 receptors is sufficient to induce unique cellular responses compared with agents blocking these receptors given alone to M-NSCLC patients (20).

Most studies conducted so far have shown that the response to ICIs in M-NSCLC patients is independent of the histological subtype (squamous or non-squamous histology) (1, 3). However, many factors contribute to the extent of the response as well as the risk of developing resistance to ICIs in these patients (1, 3). In this review, we discuss how both genetic and non-genetic intratumor heterogeneity (ITH) influences the immunogenicity of M-NSCLC, and highlight the importance of integrated genomic, pathologic and immunologic analyses to refine the selection of M-NSCLC patients who may be candidates to treatment with ICIs.

## Determinants of Response to Immune Checkpoint Inhibitors

### The Tumor Microenvironment

A key determinant of the response of M-NSCLC patients to ICI therapy is the tumor microenvironment (TME) (21–23). The TME is the ensemble of tumor cells, non-tumor cells including carcinoma-associated fibroblasts and immune cells, extra-cellular matrix as well as blood and lymphatic vessels composing a neoplastic lesion (24–27). Among malignant cells, the TME contains tumor cell subclones expressing phenotypic traits that protect them from the hosts immune system and support their ability to invade the extracellular matrix and extravasate (22, 28). On the other hand, the TME also contains a rich repertoire of tumor-infiltrating immune cells including T- and B-cells, neutrophils, dendritic cells, myeloid-derived suppressor cells, or tumor-associated macrophages, that normally constitute a natural barrier to carcinogenesis (22, 24). Inside the TME, the activity of these immune cells is strongly suppressed by cytokines, growth factors and matrix re-modelling enzymes secreted from cancer cells (22, 24, 29). These immunosuppressive effects are further intensified by the intensive aerobic glycolysis metabolism, which is observed in many tumors (22, 24–26). The TME is able to control the accumulation of T-cells inside the tumor by multiple regulatory mechanisms (30, 31). Notably, the type, density and location of immune cells within the TME play an important role in the progression of the disease and have both predictive and prognostic values in patients with M-NSCLC (31, 32). Based on the density and location of CD4 and CD8-positive TILs in the tumor center and infiltration margins of TME, tumors have been classified as “hot” when they have a high number/density of TILs or as “cold” when they contain a low number of TILs (30, 33, 34). M-NSCLCs generally fall into the “hot” category and, accordingly, patients with M-NSCLC respond relatively well to ICIs (21). However, the response is generally limited to a small subset of patients (21, 35), which is likely due to differences in the cellular composition and spatial organization of the TME, as we discuss further.

### Programmed-Death 1 and Programmed-Death Ligand 1

A key feature of M-NSCLCs that respond to ICIs is the expression of PD-1 and its ligand PD-L1 in the TILs and tumor cells of the TME (22, 29, 33). PD-L1 is expressed on the surface of tumor cells and binds to its cognate PD-1 receptor on the surface of B- and T-cells, regulatory T-cells, and NK cells (18, 19). In NSCLCs, the expression of PD-L1 protein was shown to be predictive of the response to ICIs (5, 6, 8–15). However, the expression of this protein can vary substantially between primary and metastatic lesions, as well as depending on the TME composition (2, 18). In general, M-NSCLCs are thought to be immunogenic and anti-PD-1 or anti-PD-L1 antibodies are most effective when the TME is characterized by high levels of PD-L1 expression and a high density of TILs (22, 23, 36). In the absence of TILs and positive PD-L1 expression on tumor cells, treatment with anti-PD-1 or anti-PD-L1 antibodies is expected to be less effective (23, 24, 36). One possibility is also that TILs are present in the TME, but do not express PD-1, leading to an alternative immunosuppressive mechanism (24, 36). In addition to the PD-1/PD-L1 axis, other immune regulators such as myeloid-derived suppressor cells, tumor-associated macrophages, NK cells, dendritic cells, B-cells, and various chemokine/cytokine networks operate in the TME, which likely play important roles in defining the sensitivity of M-NSCLCs to ICIs (2, 22, 24, 26).

Some challenges of the prediction of the response to ICI in M-NSCLC come from methodological variabilities, as well as, various clinically approved cut-off scores for PD-L1 expression assessment (37, 38). First of all, there is no uniformity in PD-L1 assessment among numerous clinical trials that evaluated immune checkpoint inhibitors in M-NSCLC (18, 39). Moreover, these trials used different cut-offs for considering a sample as PD-L1 positive, as summarized in **Table 1**. Notably, some studies were based on a single biopsy assessment, making the results more susceptible to intratumor heterogeneity, whereas others relied on archival tissue in which the expression might change over the time (18, 40–42). Furthermore, data on PD-L1 testing in cytological specimens, which are the predominant sample type at some institutions, are limited (43). Moreover, IHC antibodies typically bind PD-L1 at only two small hydrophilic regions that make them structurally unique and might be differentially accessible in fresh frozen *versus* Formalin-fixed paraffin-embedded (FFPE) samples (18, 39, 41). Likewise, also glycosylation of PD-L1 could cause its polypeptide antigens inaccessible to PD-L1 antibodies, which could lead to inaccurate IHC staining (44). Therefore, removal of the glycan moieties from PD-L1 to expose its polypeptide antigens has the potential to improve its detectability and to increase its utilization as a diagnostic biomarker to predict response to ICIs therapy (45).

**Table 1:** Characterization of IHC assays used for PD-L1 assessment in different clinical trials.

PD-L1 clone(species)	Company(platform)	Tested ICI(target)	Trial(no. of patients)	Cell type for PD-L1 scoring	Percentage of PD-L1 positive cells(cut-offs)	Indication
22C3 (Mouse)	Dako (Autostainer Link 48)	Pembrolizumab (PD-1)	KEYNOTE-001 (12) (495) KEYNOTE-010 (9) (1,034) KEYNOTE-024 (10) (305) KEYNOTE-021 (11) (123)	Tumor cells	TC < 1% TC ⩾ 1%, TC ⩾ 50% (min. of 100 TC)	Second-line (⩾1% of TC) First-line (⩾ 50% of TC)
28-8 (Rabbit)	Dako (Autostainer Link 48)	Nivolumab (PD-1)	Checkmate-017 (5) (272) Checkmate-057 (8) (582) Checkmate-026 (6) (541)	Tumor cells	TC < 1% TC ⩾ 1% TC ⩾ 5% TC ⩾ 10% (min. of 100 TC)	Second-line regardless of PD-L1 expression
SP142 (Rabbit)	Ventana (BenchMark ULTRA)	Atezolizumab (PD-L1)	OAK (14) (850) POPLAR (15) (287)	Tumor cells, Immune cells	TC < 1% and IC < 1% TC ⩾ 1% or IC ⩾ 1% TC ⩾ 5% or IC ⩾ 5% TC ⩾ 50% or IC ⩾ 10% (min. of 50 TC with associated stroma)	Second-line regardless of PD-L1 expression
SP263 (Rabbit)	Ventana (BenchMark ULTRA)	Durvalumab (PD-L1)	PACIFIC (13) (149)	Tumor cells	TC < 1% TC ⩾ 1% TC ⩾ 25% (min. of 100 TC)	Maintenance therapy after chemoradiotherapy (≧̸1% of TC)
73-10 (Rabbit)	Dako (Autostainer Link 48)	Avelumab (PD-L1)	JAVELIN (17) (184)	Tumor cells, Immune cells	TC < 1% TC ⩾ 1% (min. no of cells not defined)	Not approved

ICI, immunological checkpoint inhibitor; TC, tumor cells; IC, immune cells.

### Tumor Mutation Burden

In addition to the TME and PD1/PD-L1, the amount of mutations expressed by a tumor—known as tumor mutation burden (TMB)—is another major determinant of the response of M-NSCLC patients to ICIs (37, 38, 46). A summary of trials that have evaluated the association between the TMB and ICI efficacy is presented in **Table 2**. Several studies reported that TMB ≥10 mutations per megabase (mut/Mb) is predictive of longer progression-free survival (PFS) and overall survival (OS) during ICI (37, 47). In addition, the B-F1RST trial reported that TMB ≥16 mut/Mb in cell-free DNA is associated with significantly longer PFS (16). A higher TMB (>10 mut/Mb) was found in M-NSCLCs harboring driver mutations in *KRAS* or *BRAF* genes, but not in tumors with *EGFR*, *ALK*, *ROS1*, or *MET* gene mutations (3.1–6.2 mut/Mb) (48). Furthermore, adenocarcinomas were found to carry a lower TMB compared to squamous cell carcinomas (9.1 *vs.* 11.3 mut/Mb on average, respectively) (49). This observation might be explained by the fact that the etiology of adenocarcinomas is independent of tobacco exposure, making these tumors endowed with a lower neoantigen burden and therefore less immunogenic (25, 50).

**Table 2:** Summary of clinical trials that have evaluated different TMB cut-offs for predicting the response to immunotherapy.

Trial	Treatment arms	Cut-off (mutation per megabase)	No. of patients	OS		PFS	
				Median	HR	Median	HR
CheckMate 026 (6)	NIVO vs CTH	High TMB	107	18.3	1.1	9.7	0.62
		Low or medium	195	12.7	0.99	4.1	1.82
CheckMate 227 (7)	NIVO + IPI vs CTH	TMB ≥ 10	199			7.2	0.58
		TMB < 10	380			3.2	1.07
OAK (14)	ATEZO vs. CTH	TMB ≥ 10	251		0.69		0.73
		TMB ≥ 16	158		0.64		0.65
		TMB ≥ 20	105		0.65		0.61
POPLAR (13)	ATEZO vs. CTH	TMB ≥ 10	96		0.59		0.67
		TMB ≥ 16	63		0.56		0.57
		TMB ≥ 20	42		0.51		0.58
B-F1RST (16)	ATEZO	bTMB ≥ 12	22			3	0.95
		bTMB < 12	36			3.2	
		bTMB ≥ 14	14			3.4	0.73
		bTMB < 14	44			3.2	
		bTMB ≥ 16	14			9.5	0.49
		bTMB < 16	47			2.8	
		bTMB ≥ 20	8			9.5	0.23
		bTMB < 20	50			2.7	

ATEZO, atezolizumab; CTH, chemotherapy; HR, hazard ratio; IPI, ipilimumab; NIVO, nivolumab; OS, overall survival; PFS, progression-free survival; TMB, tumor mutational burden; bTMB, blood based TMB.

The TMB is typically estimated based on either whole exome sequencing (WES) or targeted sequencing (TS) of the DNA extracted from a tumor (46, 51). However, these methodologies have different sequencing coverage and depth, and therefore provide a different sensitivity and specificity in estimating the TMB (38, 46, 52). TS, which covers pre-specified small exonic or genomic regions, makes the assessment of the TMB easier, cheaper, and more practical in a clinical setting (37, 38, 46, 52). However, TS panels cover a substantially smaller fraction of the genome compare to WES probes, carrying the risk of actual TMB underestimation (53, 54). It was suggested that TS panels covering less than 300 genes or 1 Mb cause unreliable TMB results and should be avoided (54). Importantly, a crucial step in correctly estimating the TMB is the bioinformatic selection of tumor-specific single-nucleotide variants (SNVs) by filtering out germline or synonymous SNVs, which represent false positives and are unlikely involved in neoantigen generation, respectively (55, 56). Likewise to tumor-specific SNVs, also frameshift indels (small insertions and deletions) are considered a highly immunogenic mutational class that trigger an increased quantity of neoantigen, moreover, it was reported that both the SNPs and frameshift burdens are significantly associated with ICIs response (57). In addition, pre-analytical and analytical factors such as the use of FFPE samples as a source of genomic DNA, a low tumor purity or a dense TME reduce the sensitivity of TMB determination, both for TS and WES (38, 46, 47, 51).

### Neoantigens

Neoantigens are proteins with modified epitopes because of somatic mutations in their coding genes. These epitopes are loaded onto HLA molecules and displayed on the surface of tumor cells (25, 58). Neoantigens can be recognized as foreign by the host’s immune system, ultimately triggering a T-cell mediated antitumor response (50, 58). A higher TMB is expected to increase the likelihood of recognition of the tumor by neoantigen-reactive T-cells (59). In M-NSCLC patients, the co-existence of a high TMB and neoantigen expression has a positive predictive value of the response to anti-PD1, anti-PD-L1 and anti-CTLA-4 therapy (13, 60). Some studies have suggested that neoantigen heterogeneity may influence immune surveillance, however, clonal and subclonal neoantigens do not drive equally immunogenicity (13, 50, 59). Mutations induced by cytotoxic therapy enhance the subclonal neoantigens burden and might not elicit an effective antitumor response (50, 61). On the other hand, the extensive clonal mutational repertoire present in smoking-associated M-NSCLC (5) could render this disease sensitive to T-cell therapies targeting multiple clonal neoantigens, in combination with appropriate modulation of immune checkpoints (50). Likewise, the observation that the expression of neoantigens is subjected to the genetic control may have important implications for predicting the response and resistance to ICIs, and might be harnessed to develop vaccines or adoptive cell therapies (25, 58, 62). Activity of T-cells by the amount of neoantigens expressed within the tumor is regulated by the inflammatory microenvironment that controls the availability of immune-regulatory checkpoints for T-cell (22, 28, 63, 64). Tumor subclones expressing neoantigens may be preferentially eliminated by the immune system resulting in neoantigen loss (25). However, it is unclear which neoantigens are depleted as the result of the response to the therapy or tumor dissemination, and whether such phenomena only lead to tumor escape or may be harnessed to improve the response (25, 50, 58, 62).

## The Role of Intratumor Heterogeneity

In M-NSCLC, both spatial and temporal heterogeneity are considered as a main indicators of tumor diversity (65–67). The spatial type of ITH is related to discrepancies between different regions within the same tumor and may be detected at genetic and immunological level leading to a heterogeneous immune response in distinct populations of cancer cells (65, 66, 68–70). The expression of PD-1 or PD-L1 might vary considerably from region to region within the same tumor, as a result of somatically acquired genetic differences. Up to 40% of M-NSCLC patients have substantially different anti-PD-1 resistance scores in different regions of the same tumor, which often leads to discordant predictions of the extent of response to anti-PD-1 or anti-PD-L1 inhibitors (41, 70). Depending on the study, 2–46% of small biopsy samples were found to give false-negative PD-L1 expression results in comparison to surgically resected specimens (40–42, 71, 72). Moreover, in M-NSCLC, the heterogeneity of PD-L1 expression is also observed not only within the primary tumor, but also within and between coexisting metastases (40, 70–72). The metastatic sites may affect the value of PD-L1 as a predictive biomarker for ICIs treatment in NSCLC. Namely, specimens from lymph node metastases have low PD-L1 expression and are not preferred to guide ICIs treatment in clinical practice or in clinical trials (65). Among distant metastases of NSCLC, liver and adrenal sites have high PD-L1 expression, whereas it is low in brain and bones metastases (65, 73). Low PD-L1 expression in brain metastases may be related to the immune sanctuary features of this site (65, 67), whereas, bone tissues have a small pool of effective cytotoxic immune cells and a relatively large accumulation of suppressor immune cells (65, 74). This immune imbalance may favor the development of bone metastases with less selective pressure from the immune system, making PD-L1 expression in bone metastases less important for immune escape (65, 69, 74). On the other hand, liver and adrenal glands are immunologically equipped for effective tumor surveillance with potent cytotoxic T-cells and, therefore, they require inhibitory mechanisms, like up-regulation of PD-L1 expression, for cancer cells to survive (65, 69, 73).

In contrast to the spatial ITH, the temporal heterogeneity is created in between different time points during the disease course (67). The anticancer therapies may increase genetic ITH by shaping a new subclones with different somatic mutations, moreover, tumors with a highly heterogeneous subclonal structure might not produce enough neoantigens for T-cells to mount an effective anti-tumor response upon treatment with ICIs (46, 47, 50, 75). It was reported that the first line of M-NSCLC treatment may potentially affect immune response during cancer evolution leading to the response to ICIs in various ways (75, 76). In overall, chemotherapy, radiotherapy, and EGFR or ALK tyrosine kinase inhibitors increase PD-L1 expression, suggesting that up-regulation of PD-L1 is one approach that cancer cells may use to evade immune-mediated cell destruction (65, 77–79). It is worth to add that increase of PD-L1 expression after administration of the cytotoxic agents is insignificant, whereas ICIs significantly decrease the PD-L1 expression within M-NSCLC (65, 79). Today, the decision whether to administer ICIs to M-NSCLC patients is based on PD-L1 staining in primary lesions (80). However, considering the above mentioned facts that the PD-L1 status might change during treatment, all M-NSCLC patients should be re-biopsied and tested for PD-L1 expression upon therapy failure or at the time of disease progression (81).

In addition to genetic ITH, also non-genetic heterogeneity might influence the response of M-NSCLC patients to ICIs. As mentioned above, the heterogeneity of the TME can affect pathological stage, treatment efficacy and prognosis (29), and is an important predictor of antitumor response (22, 24). For example, the amount of desmoplastic stroma and the balance between promoting and inhibiting angiogenic factors (*e.g.* the vascular endothelial growth factor, VEGF) may influence the penetration of ICIs in the TME (82, 83). Additional spatial heterogeneities in the TME might cause an uneven penetration of these agents and contribute to the emergence of resistant cell populations or to the development of hypoxic niches that might support cancer stem cell phenotypes and immune evasion (21, 29, 84). Furthermore, in M-NSCLC, the tumor immune evasion capacity may be modulated at different stages of the disease either by factors stimulating the tumor immune escape or through the loss of neoantigens expression (25, 50, 62). Also spatial heterogeneity of intratumoral T-cells may be driven by the intratumoral neoantigen load and sculpted by a mutational background (25, 85).

## Conclusions and Future Perspectives

Immunotherapy based on ICIs has drastically changed the natural history of many patients with M-NSCLC. However, the path towards ensuring long-term survival to most M-NSCLC patients remains steep. Inter-patient differences in the composition and spatial structure of the TME and in the TMB influence the type and duration of response to ICIs and ultimately explain why certain patients, unlike others, have only a limited benefit from these agents. Genetic and phenotypic ITH is an important barrier limiting the effects of single-agent immune therapies. On the other hand, ITH might represent a vulnerable “Achilles’ heel” that might be targeted by combinatorial therapies and/or adaptative strategies (29, 86). More studies on ITH are needed to understand the complex interplay between tumor and immune cells and the role of spatio-temporal tumor heterogeneity in the response of M-NSCLC patients to immunotherapies. Single-cell approaches based on single-cell-sequencing or spatial transcriptomic may bring us an important step closer to understanding the role of ITH on response to ICI (87–89). Ultimately, this should lead to the development of novel therapeutic agents and/or treatment modalities, improving the prognosis of this still largely prevalent and deadly cancer.

## Author Contributions

All authors contributed to conception of the minireview. MN wrote the first draft of the manuscript and sections of the manuscript. PK, NC, and JM contributed to the draft of the manuscript revision. All authors contributed to the article and approved the submitted version.

## Funding

This work was supported by a grant from the Polish National Science Center (UMO- 2016/23/D/NZ2/02890) and a START scholarship from the Foundation for Polish Science (FNP) to MN, and Funds from Medical University of Lublin to JM.

## Conflict of Interest

The authors declare that the research was conducted in the absence of any commercial or financial relationships that could be construed as a potential conflict of interest.
